# Viral population analysis and minority-variant detection using short read next-generation sequencing

**DOI:** 10.1098/rstb.2012.0205

**Published:** 2013-03-19

**Authors:** Simon J. Watson, Matthijs R. A. Welkers, Daniel P. Depledge, Eve Coulter, Judith M. Breuer, Menno D. de Jong, Paul Kellam

**Affiliations:** 1 Wellcome Trust Sanger Institute, Wellcome Trust Genome Campus, Hinxton, Cambridge CB10 1SA, UK; 2 Department of Medical Microbiology, Academic Medical Centre, Amsterdam, The Netherlands; 3 MRC/UCL Center for Medical Molecular Virology, Division of Infection and Immunity, University College London, Cruciform Building, Gower Street, London WC1E 6BT, UK

**Keywords:** Quality Assessment of Short Read Pipeline, influenza virus, minority-variant analysis, deep-sequencing, population dynamics

## Abstract

RNA viruses within infected individuals exist as a population of evolutionary-related variants. Owing to evolutionary change affecting the constitution of this population, the frequency and/or occurrence of individual viral variants can show marked or subtle fluctuations. Since the development of massively parallel sequencing platforms, such viral populations can now be investigated to unprecedented resolution. A critical problem with such analyses is the presence of sequencing-related errors that obscure the identification of true biological variants present at low frequency. Here, we report the development and assessment of the Quality Assessment of Short Read (QUASR) Pipeline (http://sourceforge.net/projects/quasr) specific for virus genome short read analysis that minimizes sequencing errors from multiple deep-sequencing platforms, and enables post-mapping analysis of the minority variants within the viral population. QUASR significantly reduces the error-related noise in deep-sequencing datasets, resulting in increased mapping accuracy and reduction of erroneous mutations. Using QUASR, we have determined influenza virus genome dynamics in sequential samples from an *in vitro* evolution of 2009 pandemic H1N1 (A/H1N1/09) influenza from samples sequenced on both the Roche 454 GSFLX and Illumina GAIIx platforms. Importantly, concordance between the 454 and Illumina sequencing allowed unambiguous minority-variant detection and accurate determination of virus population turnover *in vitro*.

## 1. Introduction

Intra-individual RNA virus infections exist as a population of closely related virus genotypes. Current thinking states that these virus populations are mutant spectrums of genetic variants that arise through errors during genome replication that generate new genotypes. [1,2]. Owing to the often very large population sizes of RNA viruses, natural selection has a great effect in controlling the evolutionary dynamics of the virus; in combination with high mutation rates, new viral genotypes with an increased fitness are continually evolving and out-competing less-fit genotypes, oftentimes within days in response to a strong selective pressure [1,3].

The advent of massively parallel next-generation sequencing (NGS) platforms, such as Roche's GS FLX and Illumina's Genome Analyzer, allows ultra-deep sequencing to detect low-frequency minority variants within a sample. This is important for example in HIV, which exists in an individual as a population of variants at different frequencies. A strong selective pressure on the population will result in the emergence of a genotype to become the majority. This has been directly associated with disease progression (detection of CXCR4-using viruses) and precedes drug resistance [4–6]. Quantifying the virus variation within a sample may therefore have important clinical implications. However, massively parallel short reads have lower confidence for each base-call compared with capillary dideoxy-sequencing methods; the technical error rates of the 454 and Illumina platforms are approximately 0.3 per cent and 0.1 per cent, respectively [7–10]. As minority variants may be present at similar frequency as these technical errors, rigorous quality control (QC) of NGS data is essential for accurate virus genome assembly, understanding the dynamics of virus minority species and determining their clinical relevance.

Many different QC methods for NGS data have been reported [11–13], however these have limitations for analysis of short read virus genome data. For example, many work with data from only one platform, most will not de-multiplex datasets, some only quality check but do not edit the data, and some only work through a web browser on sample datasets. Here, we describe the Quality Assessment of Short Reads (QUASR) Pipeline for the processing, assessment and QC of NGS data. QUASR accepts both FASTQ files from Illumina platforms and standard flowgram format (SFF) files from 454 and Ion Torrent platforms, will de-multiplex, remove primer sequences and QC sequence data. Virus consensus-sequence and minority-variant calls from the common sequence alignment mapping (SAM)/binary alignment mapping (BAM) format are derived together with a statistical overview of the datasets. Importantly, we use both overall read and individual base quality for accurate genome assembly and minority-variant detection. By using QUASR, we show that virus genome assembly is robust and independent of the sequencing platform used. Further fine resolution minority-variant identification reveals dynamic changes in virus populations during *in vitro* culture of human influenza A/H1N1/09 virus.

## 2. Results

QUASR was implemented as described in section 7 (see the electronic supplementary material, figure S1) and assessed on a 2009 pandemic H1N1 influenza A virus (A/H1N1/09) sequentially passaged on well-differentiated human airway epithelial (HAE) cells. Four sequential viral samples were sequenced on both the Roche Genome Sequencer FLX 454 and Illumina's genome analyzer IIx. All primer-removed and quality-assured read-sets were mapped with SSAHA v. 2.5.3 [14] to the A/H1N1/09 reference sequence A/California/04/2009. From the resulting SAM file, a pileup file was created using SAMtools v. 0.1.17 [15]. This was then parsed by QUASR to create quality-dependent consensus-sequences. Using the A/H1N1/09 data, we empirically assessed the consequence of altering filtering parameters on virus genome assembly and accurate minority population assessment.

## 3. Impact of quality parameters on viral genome short read sequences

**Figure 1.**
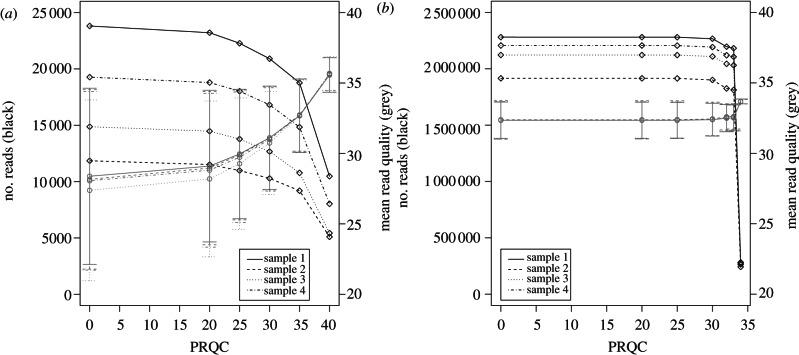
(*a*) Read numbers (black line) and mean ‘mean read-quality-score’ (grey line) with error bars indicating standard deviation about this mean for the four 454 read-sets and (*b*) four corresponding Illumina read-sets. Increasing the per-read quality score (PRQC) cut-off has a marked effect on 454 read-sets, discarding up to 59% of reads and resulting in a nearly 6-fold increase in base-calling confidence. PRQC has a negligible effect on Illumina read-sets up to 34, where 88% of the reads are discarded for a 1.35-fold increase in confidence.

We derived read quality data relating to read length, read median quality and per-base quality scores along the read length for use in the assessment of quality-based filtering metrics (see the electronic supplementary material, figure S2). In particular, we used QUASR to trim low-quality bases to maximize the ‘per read’ median quality and read length (see the electronic supplementary material, figure S2*c*, upper right quadrant). From the primer-removed read-sets for each sample, we determined the effect of filtering based on median per-read quality cut-offs (PRQCs) for the 454 (figure 1*a*) and Illumina (figure 1*b*) read-sets. Prior to PRQC, the mean quality score of the 454 read-sets was 28±6.35, whereas the mean of the Illumina read-sets was 32.36±1.33. At a PRQC of 40, an average of 58.7 per cent of reads was discarded from the 454 read-sets, resulting in a mean quality score of 35.61±1.21. For Illumina read-sets, a steep drop-off in read numbers is seen at PRQC34, with 87.5 per cent of the reads being discarded, resulting in a mean quality score of 33.68±0.17. However, at PRQC33, an average of 4.62 per cent of the Illumina reads were discarded, with a resultant mean quality score of 32.55±0.91. PRQC therefore trims and discards an insignificant number of Illumina reads up to a PRQC of 33, and results in little overall increase in mean quality score. By contrast, for 454 read-sets, PRQC has a greater effect, with a mean increase in the quality of 7.61 by PRQC40. As this quality increase is *phred*-scaled, the confidence of correct base calling is increased 5.77-fold for 454 read-sets but at the cost of discarding an average of 58.7 per cent of reads (figure 1*a*). Overall, high-quality reads of equivalent per read quality can be derived from both 454 and Illumina platforms.

## 4. Assembling error-free consensus-sequence influenza genomes from 454 and Illumina reads

**Figure 2.**
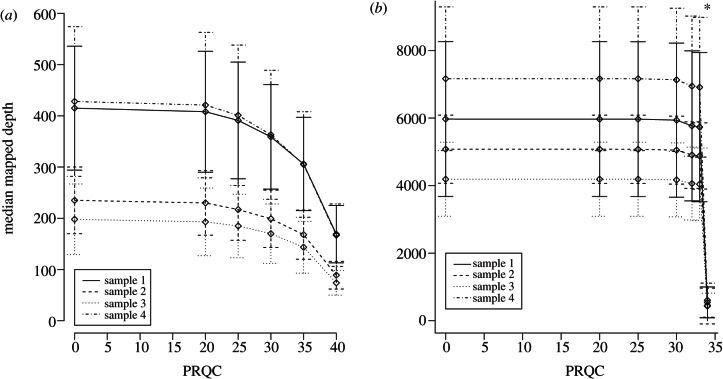
(*a*) Median coverage depth for the 454 read-sets and (*b*) the Illumina read-sets with error bars indicating the median absolute deviation (MAD) about their median. The asterisk over PRQC34 indicates that genome coverage dropped below 100%. (*a*) The trends match that of figure 3*a*, with the mapped depth dropping by 61% to 125-fold coverage at PRQC40. The mean MAD decreases from 100 to 42, indicating that the majority of the discarded reads are from regions of high coverage. (*b*) Illumina's mapped depth trend mirrors that of the read numbers too; at a PRQC of 34 mapped depth drops by 91%, resulting in incomplete genome coverage. Therefore, PRQC values of 40 and 33 are used for subsequent 454 and Illumina analyses, respectively.

We assessed how different read filtering parameters affected the final number of mapped reads (table 1) for genome assemblies for each sequencing platform by mapping each quality-controlled read-set as described above. For 454, full genome coverage is retained even at a PRQC of 40, with a median mapped depth of 125 (figure 2*a*). However, at the Illumina read-sets’ maximum PRQC value (PRQC34), only 97 per cent genome coverage is attained, with an average median-depth-coverage of 509. At PRQC33, full genome coverage is retained by all samples, with an average median-depth-coverage of 5391. Therefore, for all subsequent analyses, we used PRQC values of 40 and 33, and length cut-offs of 150 and 50 for 454 and Illumina read-sets, respectively, where the mean 454 read length before processing was 462.95 bp (range 97.75–703.25) and for Illumina reads was 54 bp paired-end.

**Table 1.** Read and mapping statistics for the four serially passaged A/H1N1/09 samples co-sequenced on 454 and Illumina

sample	platform	reads pre-QC (×10^4^)	reads post-QC (×10^4^)	read length pre-QC	read length post-QC	mean mapping quality pre-QC	mean mapping quality post-QC	median mapped depth
1	454	2.38	1.05	463.63	367.97	178.21	199.43	169
	Illumina	228.09	218.24	54.00	53.98	47.37	47.33	5733
2	454	1.19	0.51	461.64	361.48	150.84	162.52	89
	Illumina	191.56	181.19	54.00	53.97	46.37	46.34	4879
3	454	1.49	0.54	462.73	345.85	142.81	159.09	74
	Illumina	212.23	203.11	54.00	53.98	43.28	43.22	4041
4	454	1.93	0.80	463.81	359.82	151.21	165.89	167
	Illumina	220.68	210.64	54.00	53.98	47.23	47.21	6912.5

**Figure 3.**
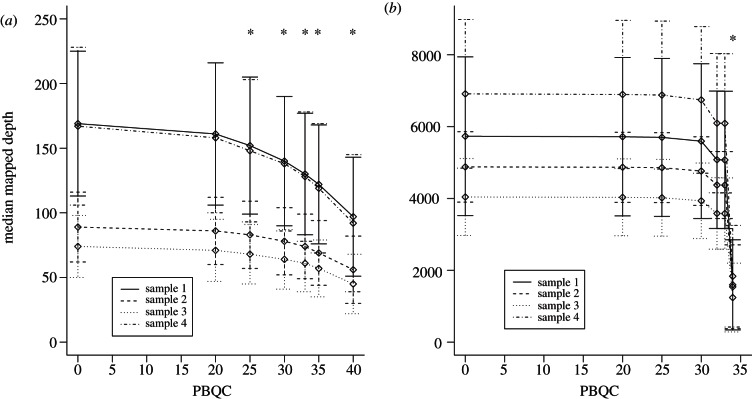
(*a*) Median mapped coverage depth for the 454 read-sets and (*b*) the Illumina read-sets at increasingly stringent per-base quality control (PBQC) values, and a PRQC of 40 for 454 and 33 for Illumina. Asterisks indicate where genome coverage drops below 100%. (*a*) Full genome coverage is retained up to PBQC20, beyond which gaps appear in the genome at homopolymer stretches and regions of low coverage. (*b*) An 11% decrease in median mapped coverage is observed at PBQC32, while still retaining full genome coverage. Little difference is seen by increasing this to 33, but at 34 the majority of the bases are removed, resulting in incomplete genome coverage.

Treating complete reads for quality assessment nevertheless masks the effect of individual low-quality positions within a read. Although such positions are reported to be randomly distributed, evidence is accumulating that this may not always be the case and as such low-quality nucleotides within a read may accumulate in certain genome locations [8]. This can affect consensus generation and minority-variant detection. Consequently, such low-quality positions should be identified and masked when generating consensus-sequences and calculating minority variants. We therefore assessed the effect of different per-base quality cut-offs (PBQCs) on mapped read depths and minority-variant prediction. To determine the optimal value to apply to these data, we applied the same increasingly stringent cut-offs to the mapped data as with PRQC (figure 3). As expected, the PBQC parameter had a pronounced effect on the 454 read-sets, with gaps appearing at the ends of homopolymeric tracts at PBQC25 owing to the decreased confidence assigned by the 454 base-caller at homopolymers. For Illumina read-sets, the PBQC parameter had a greater effect than the PRQC cut-off, with an 11 per cent decrease in median mapped depth at PBQC32. Increasing the threshold value to 34 removed 71 per cent of bases, but resulted in incomplete genome coverage. Therefore, PBQC values of 20 and 33 were chosen for subsequent 454 and Illumina analyses, respectively, and applied to the read-sets post-mapping by the consensus generation and minority-variant determinant scripts. For consensus-sequence generation, the ambiguity frequency parameter, which sets the minimum frequency at which a minority base must be present to be included in the consensus as an ambiguity code, was set to 0.5.

## 5. Consistent minority-variant detection following quality filtering of 454 and Illumina read-sets

A/H1N1/09 consensus-sequences generated without any QC imposed on them were compared against those with the QC settings described in section 4. The unfiltered read-sets showed differences between the 454 and Illumina consensuses in three out of the four samples (table 2). Applying the QC parameters described above resulted in a reduction in the number of differences, with two out of the four samples being identical across the entire genome. All ambiguity differences seen in the unfiltered consensus-sequences were resolved correctly; however, in sample 2, two new differences appear between the 454 and Illumina assembled genomes following QC (NA A920G and NA G1382A). These are due to low coverage in the 454 sample in this segment following QC, resulting in insufficient representation of the base profile at these positions. Therefore, by assigning a final control parameter, namely a minimum coverage threshold, we can eliminate all differences between the two sequencing platforms on the same samples.

**Table 2.** Differences between consensus-sequences for four serially sampled influenza samples sequenced on 454 and Illumina platforms, before and after QC.

segment	sample							
	1		2		3		4	
	pre-QC	post-QC	pre-QC	post-QC	pre-QC	post-QC	pre-QC	post-QC
PB2					T252Y			
					A792R			
PB1					C159Y			
			A1710R					
NA				A920G				
				G1382A	G1382R			
HA					R536G		R536G	
			A861R					
NP					C282A	C282A		
			A1419R					
total	0	0	3	2	6	1	1	0

**Figure 4.**
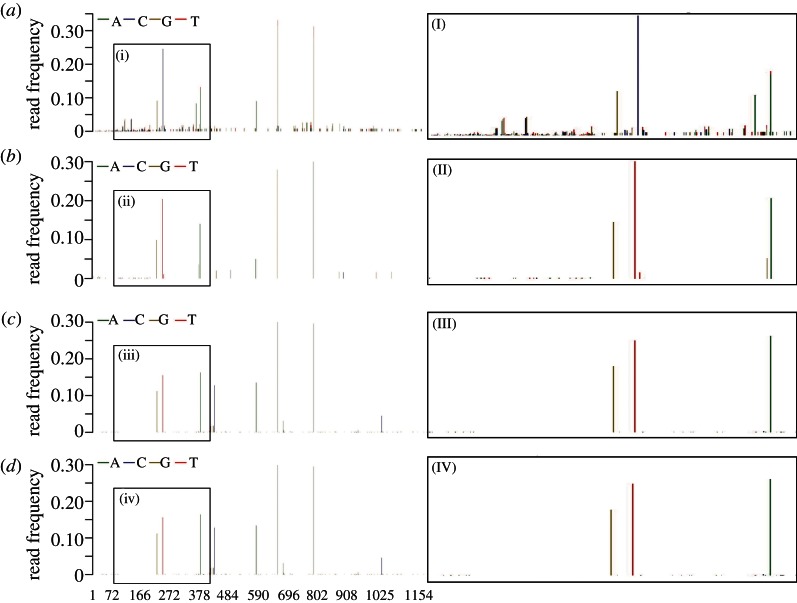
(*a*) Minority base frequencies for PB2 segment of sample 3, sequenced on the 454 platform without QC and (*b*) following PRQC40 and PBQC20, and (*c*) on the Illumina platform without QC and (*d*) following PRQC33 and PBQC33. Each minority base profile has the region between nucleotides 72 and 378 expanded for clarity. Performing QC on the 454 samples removes the many low-frequency (≤0.01) low-quality variants (I,II), thereby removing technical errors; the fewer remaining minority variants have greater support for further investigation. Importantly, some minority variants present at frequencies ≥0.1 before QC disappear following QC, and some that appear to be insignificant become significant afterwards. The minority-variant profiles following QC look much similar between the two platforms following QC (II) and (IV) than before (I) and (III).

Minority-variant profiles were calculated for each read-set under the QC parameters defined above. These were compared against the profiles of the unfiltered samples to determine the effect of QUASR on the minority variants (figure 4). The effect on the 454 read-sets is pronounced (figure 4*a,b*), with many of the low-frequency (less than 2%) minority bases being removed, resulting in a minority-base profile much more consistent with that of Illumina (figure 4*c*). Importantly, QC addressed cases where higher-frequency minority bases differed between 454 and Illumina (I and III in figure 4). Therefore, QC-based parameters used here address the systematic base miscalls that might otherwise influence the minority frequencies at that position.

**Figure 5.**
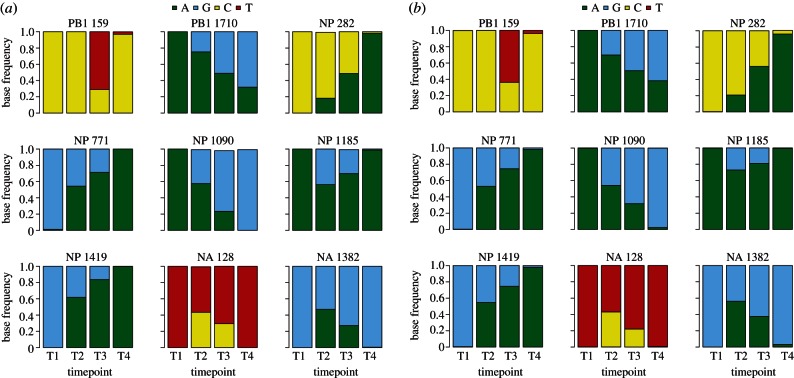
(*a*) Base frequencies displayed as a stacked histogram for nine different genome positions sequenced on 454 and (*b*) Illumina. For each position, the base frequencies are shown across four time points, allowing the dynamics of the minorities at that position to be observed. There is little difference in the base frequencies between the two platforms; the dynamics are almost identical, indicating platform independence in observing the changes in minorities, despite their technological differences. Two different population dynamics can be observed in the time series; five of the positions have their majority base replaced while the other four show a transient appearance of minority variants that are subsequently lost.

To formally assess the ability to detect changes in virus minority species over time, we compared the time-dependent changes in minority variants across the whole influenza genome in the four sequential samples serially passaged *in vitro* for both 454 and Illumina read-sets (figure 5). Nine positions in three different gene segments were found to vary over the passage series. Variants present at 1 per cent at one time point (NP771, NP1090, NP1419) were completely replaced at later time points. Interestingly, patterns of nucleotide change suggest two different population dynamics. PB1 1710, NP 282, NP 771, NP 1090 and NP1419 are all completely replaced over the time course, whereas PB1 159, NP 1185, NA 128 and NA 1382 show a transient appearance of minority variants that are then lost. Importantly, following QC procedures outline here, the magnitude and time of each genome position change is essentially the same for both 454 and Illumina sequencing read-sets.

## 6. Discussion

A prerequisite for large-scale, next-generation sequence analysis of virus genomes and assessment of intra-host virus genome diversity is the consistent processing, assembly and analysis of short read data. Here, we described QUASR, designed for virus genome analysis using NGS, which processes raw base-call data from 454 and Illumina platforms using defined QCs and post-assembly analyses. The code is freely available (http://sourceforge.net/projects/quasr) and is memory-efficient, removing the need for large computing resources.

We applied QUASR initially to four serially sampled influenza A/H1N1/09 datasets generated on both 454 and Illumina platforms. As expected, there is a more pronounced effect of QUASR pre-processing on 454 than Illumina reads. This is because of the intra- and inter-read variation seen in the 454 read-set, with reads ranging in size from less than 100 to over 700 bp, and *phred-*scaled qualities following a distribution between 1 and 40. Illumina read-sets, on the other hand, have a uniform read length, and their qualities scores primarily range between 31 and 33. The 3′-end of 454 reads also have significant drop-off in quality, which makes QUASR's 3′-trimming algorithm highly effective at increasing a read's median quality.

Despite the technological differences between the 454 and Illumina platforms that result in different error profiles and require different assembly strategies, we show that there is little difference in the resultant consensus-sequences and minority-variant frequencies following QC. However, overly stringent filtering thresholds can adversely affect the consensus. This is particularly true of 454 datasets, where reduced read depths can result in insufficient sampling of the population's base profile at a given position. For this reason, QUASR includes read depth information by marking in lowercase positions in the consensus-sequence that are low coverage areas of the assembly.

Minority base profiles of a sample differ between platform more than the consensus-sequence, with non-QC 454 read-sets showing many more low-frequency technical errors. By performing QC on these data, these technical errors disappear, resulting in much greater agreement between the profiles of the two sequencing platforms. Importantly, this removes the tendency for systematic sequencing errors being perceived as biologically interesting higher-frequency variants in the absence of such QC. The power of short read NGS comes from the increased read depth at which a sample can be sequenced. In order to fully use this power, it is imperative that the data be properly and consistently quality-controlled with software such as QUASR, and for QC parameters to be recorded preferably with the genome sequences to maximize the confidence in the underlying consensus genomes and minority base frequencies. This will be essential as such sequencing methods and the assessment of minority variants becomes routine for analysis of clinical samples to inform public health, pathogen surveillance and treatment strategies. Furthermore, with the continued development of different algorithms for processing, quality-controlling and analysing virus genome NGS datasets, we suggest that a formal comparison of the various software will be essential.

## 7. Methods

### (a) Sample preparation

Influenza samples were collected during an *in vitro* serial passaging experiment in which HAE cell cultures were infected with a clinical sample obtained from a patient with a proved A/H1N1/09 infection. Samples were collected during peak infection of the epithelial cell cultures (generally between 24 and 48 h after infection), and viral loads were determined using a Taqman assay in order to rule out low copy input. RNA was isolated using a MagnaPure automated extractor, and RNA was RT-PCR amplified with the One-Step SuperScript III RT-PCR kit (Invitrogen) using a modified eight-segment PCR method [16]. In brief, three RT-PCRs were performed for each sample, using primers common-uni12 (5′-GCCGGAGCTCTGCAGATATCAGCRAAAG CAGG-3′), common-uni12G (5′-GCCGGAGCTCTGCAGATATCAGCGAAAGCAGG-3′) and common-uni13 (5′-CAGGAAACAGCTATGACAGTAGAAACAAGG-3′). The first RT-PCR contained the primers at the following final concentrations: 0.2 µM common-uni12 and 0.2 µM common-uni13. The second RT-PCR contained the primers at the following concentrations: 0.2 µM common-uni12G and 0.2 µM common-uni13. The latter PCR greatly improved the amplification of the PB2, PB1 and PA segments. The third RT-PCR is a control PCR in which the reaction mix is identical to the first RT-PCR but without the addition of reverse transcriptase, but only Platium Taq HiFi polymerase to exclude the presence of any influenza amplicon contaminant. Reactions were performed in a volume of 50 µl, and contained 5.0 µl RNA, and final concentrations of 1×SuperScript III one-step RT-PCR buffer, 0.2 µM of each primer and 1.0 µl SuperScript III RT/Platinum Taq high fidelity enzyme mix. Thermal cycling conditions were: reverse transcription at 42°C for 15 min, 55°C for 15 min, 60°C for 5 min; initial denaturation/enzyme activation of 94°C for 2 min; five cycles of 94°C for 30 s, 45°C for 30 s, slow ramp (0.5°C s^−1^) to 68°C, 68°C for 3 min; 30 cycles 94°C for 30 s, 57°C for 30 s, 68°C for 3 min; and final extension of 68°C for 5 min. Finally, equal volumes of both reactions were combined producing a near equimolar mixture of all influenza segments and total DNA concentration determined using the Quant-iT PicoGreen dsDNA Picogreen assay (Invitrogen).

### (b) Library preparation and Illumina sequencing

Products from the eight-segment PCR with a DNA concentration of approximately 50 ng µl^−1^ were sheared to lengths of between 200 and 400 bp using a Covaris AFA (Covaris, Woburn, MA, USA), end-repaired, A-tailed and ligated with Illumina sequencing adaptors containing identifying tags allowing multiplex sequencing of 12 samples per lane. The products were sequenced on an Illumina GAIIx using a paired-end 54 bp run, following the manufacturer's instructions. All generated data were deposited in the European Nucleotide Archive under accession nos ERS180793–ERS180796.

## 8. Outline of quality assessment of short read

Reads generated by the 454 and Illumina platforms were processed using QUASR version 6.08. QUASR was written in Python3, with graphical output requiring the R software suite [17]. QUASR was specifically designed with a low memory-footprint in mind, allowing the user to parse large read-sets on local machines rather than requiring access to large-memory computing clusters, while minimally slowing down the parsing of read-sets. It was also designed to be generic, so it can be applied to any NGS dataset that generates SFF or FASTQ files. QUASR consists of two pre-assembly scripts written to prepare raw data for assembly, and a post-assembly suite of scripts for analysing the assembled data. Duplicate removal was not performed on the dataset, but is also an option within QUASR; reads are considered duplicates if they have the exact same sequence and length. If duplicates are found, then the read with the highest mean quality score is retained. For paired-end datasets, both the forward and reverse mates must be identical to be removed. QC is achieved through a two-step filtering process: prior to assembly, reads are filtered and discarded on their median quality score. Post-assembly, individual bases are ignored if their quality score is below the threshold. It should be noted that these QC steps only consider the quality scores during filtering, and do not compare the sequences of overlapping forward and reverse reads at a position. Therefore, users should be mindful of sequence-specific errors that may show up as variants. A README file describing the different input parameters and generated output files is included with the source code package.

## 9. Pre-assembly scripts

### (a) Data processing

An overview of the two pre-assembly scripts is given in electronic supplementary material, figure S1. The data-processing script will accept both Sanger-encoded and Illumina-encoded FASTQ files, as well as SFF files generated by the 454 and Ion Torrent systems. SFF files are converted into the FASTQ format and, all subsequent processing steps are performed on reads in the FASTQ format. If the input file contains multiplexed samples, the reads associated with the relevant multiplex identifiers (MIDs) can be split into separate FASTQ files. This de-multiplexing can occur in one of two ways: if the MID index tags are present in the read sequence, then QUASR will excise the tags and split the reads according to the MID associated with that index, otherwise it will look for the MID number in the read's header. For non-standard MID sequence tags, an optional file containing the mapping between the MID number and sequence can be parsed.

In addition to de-multiplexing, the data-processing script optionally excises non-biological primer sequences from reads. By providing a file containing the primer sequences, each read in turn is compared against the primer sequences, and if a match is found, then all nucleotides up to the matched sequence are excised from the read.

Quality-assurance (QA) graphs, giving a statistical overview of the read-set, can be generated through the R software suite (see the electronic supplementary material, figure S2). These give a visual representation of the distributions of read-length, GC% and median read quality in the read-set as well the cross-sectional average drop-off in base quality at the 3′-end of the reads.

### (b) Per-read quality control

The second pre-assembly script performs QC on the read-set and can be run independently of the data-processing step, only requiring that the input files be in FASTQ format. The core of the QC algorithm (see the electronic supplementary material, figure S1) iterates over the reads in the read-set, and for each read, it compares the read length against a length-cut-off value. If the read is shorter than the cut-off, then it is discarded. Otherwise, the read's median quality score is calculated and compared against a median cut-off value. If the median quality is greater than the cut-off, then the read is written to the output FASTQ. If the median quality is less than the cut-off, then the algorithm iteratively trims the 3′ base and recalculates the median quality until either the read length falls below the length-cut-off, or the median quality is raised above the median cut-off. The algorithm trims from the 3′ end of the read because the confidence of base-calling in all current platforms is significantly lower in later cycles owing to phenomena such as lagging-strand dephasing [8,18]. The reason the read is assessed on its median quality score is to make it robust to outliers; an otherwise high-quality read with a single very low-quality base will not be rejected, meanwhile the low-quality base will still be filtered out in the post-assembly quality check. This way, QUASR maximizes its use of the high-quality information in the dataset.

This QC can be performed on paired-end sequenced read-sets—both mates have to pass the QC for the reads to be written to the output files. This is to ensure that each read always has a mate, which some assemblers require for paired-end mapping. As this QC algorithm acts on reads as a whole, it is referred to as PRQC to differentiate it from the masking of individual bases performed by the post-assembly scripts, accordingly referred to as PBQC. In order to provide full control over the extent of PRQC performed on the dataset, the user specifies the length- and median cut-offs. QA graphs can be produced post-QC, allowing for visual identification of how the PRQC has affected the read-set.

## 10. Post-assembly scripts

In addition to the pre-assembly scripts, QUASR is packaged with a suite of scripts for generating majority consensus-sequences, and for determining and visualizing minority variants, from the pileup assembly file. The pileup format can be generated by the SAMtools software package or the genome analysis toolkit [19] from the SAM or associated BAM format output by most common assemblers (SSAHA2, SMALT, BWA, Bowtie).

### (a) Consensus generation

QUASR's consensus-sequence generation script has three parameters that give the user control over how the consensus is generated. The first of these is the ambiguity threshold, which allows the user to specify the frequency at which a minority base must be greater than to be included in the consensus-sequence as an ambiguity code. This gives control over the sensitivity of the consensus-sequence to minority populations. Second is the PBQC, which filters out all bases whose quality score falls below a cut-off. This reduces the number of systematic errors [8], thereby improving the confidence in the consensus-sequence and reducing the number of ambiguities owing to low-confidence bases. The third parameter is the depth cut-off; positions with a read depth less than the specified cut-off are written in lowercase in the consensus-sequence, allowing unreliable consensus-base calls to be identified easily. These three cut-offs allow much more information on the quality of the mapped read-set to be included within the consensus-sequence.

### (b) Minority-variant determination and visualization

Through R, QUASR will generate stacked histogram plots of the minority bases across the genome. The frequency of each base is calculated for each genomic position, and the most frequent base discarded. The remaining bases are then plotted as a stacked histogram, giving a visual overview of the differences to the majority sequence in the assembled read-set. Additionally, a list of the genomic positions that contain a minority base greater than a user-specified frequency can be written to an output file along with the consensus bases at each position. As with the consensus-sequence generation script, a minimum quality score cut-off can be specified, allowing the user to disregard the less-confident base calls, thereby reducing the number of false positive minority bases. A depth cut-off parameter will also exclude positions with low coverage, as their minority frequencies will be skewed owing to insufficient sampling.
